# MS26/CYP704B is required for anther and pollen wall development in bread wheat (*Triticum aestivum* L.) and combining mutations in all three homeologs causes male sterility

**DOI:** 10.1371/journal.pone.0177632

**Published:** 2017-05-16

**Authors:** Manjit Singh, Manish Kumar, Katherine Thilges, Myeong-Je Cho, A. Mark Cigan

**Affiliations:** DuPont Pioneer, Johnston, Iowa, United States of America; USDA Agricultural Research Service, UNITED STATES

## Abstract

Development of anthers and pollen represents an important aspect of the life cycle in flowering plants. Genes contributing to anther and pollen development have been widely studied in many plant species. *Ms26/CYP704B* genes play an important role in pollen development through biosynthesis of sporopollenin for pollen exine formation. To investigate the role of *Ms26/CYP704B* genes in anther and pollen development of bread wheat, mutations in the A-, B-, and D-homeologs of the putative *Ms26*/*CYP704B* gene were analyzed. Single and double homozygous mutants in any of the homeologs did not affect pollen development and male fertility. Triple homozygous mutants resulted in completely male sterile plants that were defective in pollen and anther development. Additionally, double homozygous-single heterozygous mutants were also male sterile although with varying levels of residual fertility. The fertility of these triple mutants was dependent upon the homeolog contributing the wild-type allele. Two heterologous *Ms26*/*CYP704B* genes, when transformed into a triple homozygous mutant background, completely restored male fertility, whereas a single gene was unable to restore fertility. Functional analysis of *Ms26*/*CYP704B* furthers the understanding of male fertility genes which can be utilized for the development of novel hybrid seed production systems in wheat.

## Introduction

Flowering plants have developed specialized structures for the production of male and female gametes. Successful production of male gametes relies on proper formation of male reproductive organs. Pollen grains (microgametophytes) are formed by the anther, the male reproductive organ, and deliver male gametes to organs bearing female gametes. Pollen grains are surrounded by protective pollen walls, intine and exine, to enable survival of pollen in what are often adverse environmental conditions. The intine is composed of cellulosic material whereas the major component of exine is sporopollenin (reviewed in Quilichini et al. [1]). Sporopollenin fortifies the exine as the foundation of a skeletal structure as well as through formation of a durable covering. The components of the exine are synthesized by the surrounding tapetum and deposited on the surface of developing microspores within the anther locule [2], whereas the components of the intine are believed to be generated by the microspore vegetative cell [3]. Pollen exine in rice consists of two layers, the tectum (sexine and baculae) and the nexine (foot-layer). Exine development starts with the formation of primexine at tetrad stage (reviewed in Li and Zhang [4]). Primexine, a microfibrillar matrix composed mainly of cellulose, serves as a template for deposition of sporopollenin precursors. Following the release of microspores from tetrad, the tectum is formed on the primexine through deposition of sporopollenin precursors. With the progression in development, the sporopollenin is gradually deposited to thicken and consolidate the exine. Although a detailed biochemical analysis of sporopollenin has proven difficult, it is known to contain phenolics and polyhydroxylated aliphatics, covalently coupled by ether and ester bonds [5–8].

Recently, significant advances have been made in understanding the genes involved in pollen exine formation, including sporopollenin biosynthesis, in *Arabidopsis* and rice (reviewed in Zhang et al.[9]; Gomez et al.[10]). Several genes involved in synthesis of fatty acid precursors of sporopollenin have been identified [11]. Subfamilies CYP703A and CYP704 of cytochrome P450s have an essential role in hydroxylating the fatty acid constituents of predicted sporopollenin precursors. CYP703As catalyze the in-chain hydroxylation of fatty acids and heterologous CYP703A2 protein from *Arabidopsis* can catalyze in-chain hydroxylase of fatty acids with chain length from C10 to C16 [12]. Conversely, rice CYP703A3 has been shown to hydroxylase only lauric acid, preferably generating 7-hydroxylated lauric acid [13]. CYP703A3 in rice is required for the development of anther cuticle and pollen exine. The *Arabidopsis* CYP704B1 catalyzes the in-chain and ω-hydroxylation of fatty acids and is essential for exine biosynthesis [14]. Pollen from *cyp704b1* mutant plants lack normal exine, but remain viable and capable of fertilization [14]. The rice *OsCYP704B2* gene encodes a long-chain fatty acid hydroxylase capable of metabolizing very similar substrates *in vitro* as CYP704B1. Pollen grains in *cyp704B2* mutants also lack a detectable exine resulting in male sterility. Anthers in these mutants have a defective tapetal layer and undeveloped cuticle [15]. The maize *Ms26* gene encodes a cytochrome P450 mono-oxygenase enzyme (CYP704B1) [16, 17] and microspores in *ms26* mutants have a defective exine characterized by lack of sporopollenin deposition [18]. Similarly, sorghum mutants lacking functional MS26/CYP704B are male sterile due to defects in microspore development [19].

The conserved role of MS26/CYP704B in different species provided an opportunity to investigate its role in wheat. Bread wheat (*Triticum aestivum* L.) is an allohexaploid (2n = 6x = 42) combining ancestral genomes of *Triticum urartu*, *Aegilops speltoides*, and *Aegilops taushii*, contributing the A-, B-, and D-genomes, respectively [20, 21]. Gene expression in bread wheat is often characterized by asymmetrical contribution of the three homeologous genomes as a result of genetic and epigenetic changes following polyploidization [22–24]. Functional analysis of the homologs of *Ms26/CYP704B* genes can be important for understanding the role of these genes in wheat reproductive development. Generation of multiple recessive mutant alleles in the wheat homeologs of *Ms26/CYP704B* gene through a custom-designed homing endonuclease was previously reported [19]. In this study, functional and cytological analyses of these mutants and their combinations were performed. Results indicate the importance of all three homeologs towards male fertility albeit with underlying differences. Apart from elucidating the function of male fertility genes, *Ms26/CYP704B* mutant alleles provide a novel source of nuclear male sterility in wheat that can be utilized for the development of hybrid seed production systems and exploitation of heterosis.

## Results

### Homeologs of *TaMs26* are highly similar to grass *Ms26/CYP704B* genes and are specifically expressed in anthers

**Fig 1:**
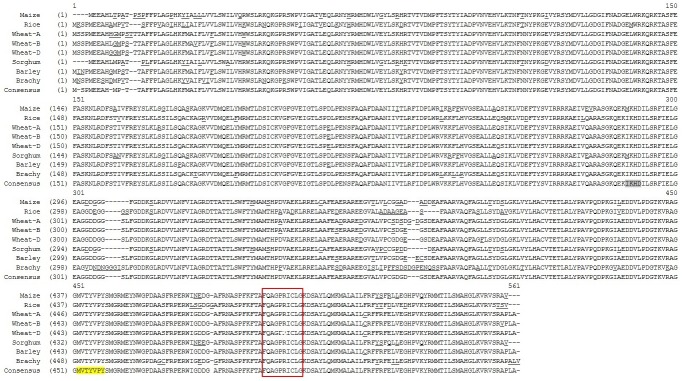
Comparison of MS26 protein sequence across grass species. Sequence differences in rice (XP_015629295.1), wheat (A-, B-, and D- genomes), sorghum (XP_002465796), barley (BAK08270), and *Brachypodium* (Brachy; XP_003558727.1) as compared to maize MS26 protein (NM_001137176) are indicated as underlined amino acids. A gap in sequence is indicated by a hyphen (‘-’). Within the consensus sequence, sequence corresponding to the 5’ end of exon 4 and the Ems26+ recognition site is highlighted in gray and yellow, respectively. The haem-binding loop is sequence boxed in red.

To compare the wheat genes homologous to *Ms26/CYP704B*, genomic sequences corresponding to the three homeologs were obtained through BLAST (Basic Local Alignment Research Tool; [25]) of the TGACv1 wheat genome assembly (*T*. *aestivum* cv. Chinese Spring) (http://plants.ensembl.org/Triticum_aestivum/Info/Annotation) with maize *Ms26* mRNA sequence (accession NM_001137176). Sequence analysis showed that each of the wheat homeologs have four predicted exons and three introns with a combined exon-intron length of 1926 bp for A-genome and 1920 bp for B- and D-genomes (S1 Fig). These genes represent coding DNA sequences (CDS) of 1665 bp for A-genome homeolog, and 1656 bp for B- and D-genome homeologs, which are capable of encoding for proteins of 554 and 551 amino acids in length, respectively. Comparison of the predicted amino acid sequences showed that the three homeologs are highly similar with 98–99% amino acid identity (Fig 1). The wheat Ms26/CYP704B proteins also show high similarity (96–97% identity) to putative Ms26/CYP704B from barley (BAK08270), while identity with Ms26/CYP704B proteins from rice (XP_015629295.1), maize (NP_001130648.1), sorghum (XP_002465796), and *Brachypodium* (XP_003558727.1) was 88–89% (Fig 1). Given the high sequence identity of the wheat genes to other grass genes orthologous to the *Ms26/CYP704B* maize and rice genes, wheat *Ms26/CYP704B* from here onwards is denoted as *TaMs26;* and *TaMs26* homeologs for A-, B-, and D-genome are denoted as *TaMs26*-*A*, *TaMs26*-*B* and *TaMs26*-*D*, respectively.

**Fig 2:**
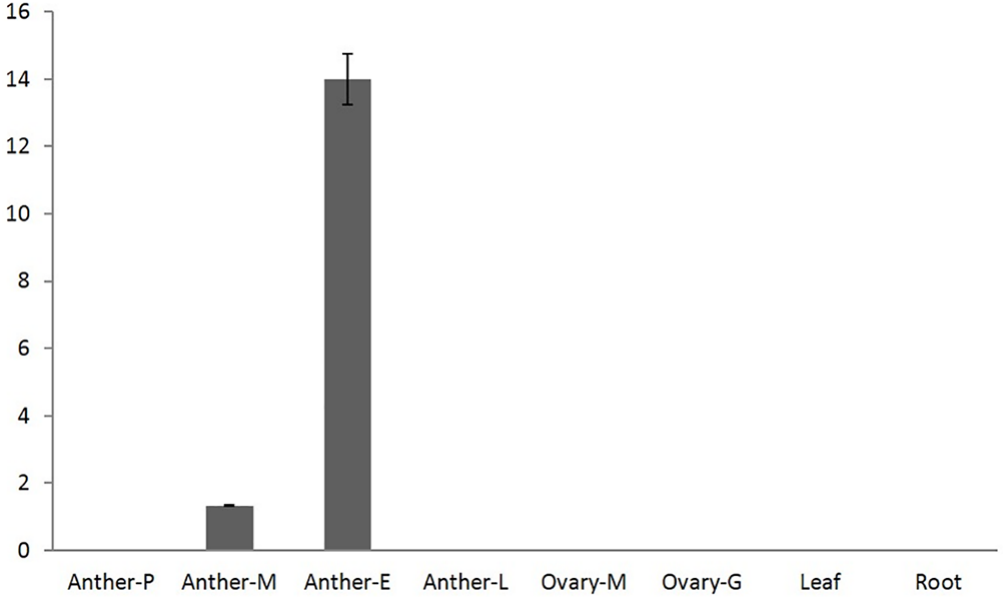
Spatial and temporal expression of *TaMs26* determined by qRT-PCR. Anther and ovary stages correspond to P, pre-meiosis; M, meiosis II; E, early uninucleate; L, late uninucleate; G, gametogenesis. Profiles were determined relative to *TaActin* expression. Error bars indicate SE. The y axis shows arbitrary units.

To study the RNA expression of the wheat *TaMs26* homeologs, RT-PCR was performed with primers located in Exon 4 sequence homologous to all three genomes. qRT-PCR and semi-quantitative RT-PCR revealed that the wheat *TaMs26* homeologs are expressed in the anthers from tetrad (late meiosis II) to early uninucleate microspore stages (Fig 2 and S2 Fig) with maximum expression at the uninucleate microspore stage. No expression was detected in the anthers at pre-meiotic and late uninucleate microspore stages or in the ovary, leaf, and root tissues. To estimate the distribution of RNA transcripts from each homeolocus, Illumina deep sequencing was performed on the RT-PCR product from the early uninucleate stage. The number of sequence reads obtained corresponding to *TaMs26*-*A*, *TaMs26*-*B*, and *TaMs26*-*D* was 256,228 (33%), 282,829 (37%), and 231,026 (30%), respectively, out of 770,083 sequence reads; thus indicating that all three homeologs were expressed in the anthers at comparable levels.

### Two wild-type alleles of *TaMs26* are required for maintenance of male fertility

To analyze the functional contribution of *TaMs26* to pollen development in wheat, previously generated single recessive mutant alleles in the three homeologs [19] were utilized. For simplicity, homozygous mutations in A-, B- and D-homeologs are denoted as *TaMs26-a*, *TaMs26-b* and *TaMs26-d*, while heterozygous mutations are denoted as *TaMs26-Aa*, *TaMs26-Bb* and *TaMs26-Dd*, respectively. Plants containing five non-identical mutations, i) 4 bp Δ in *TaMs26-A*, ii) 23 bp Δ in *TaMs26-B*, iii) 81 bp Δ in *TaMs26-B*, iv) 90 bp Δ *TaMs26-D*, and v) 96 bp Δ + 2 bp I in *TaMs26-D*, were used for crossing experiments and further analysis. To track the different alleles, PCR assays with genome-specific primers were designed to genotype mutations in *TaMs26-A*, *TaMs26-B*, and *TaMs26-D* (see Materials and methods). Previous analysis of single mutants showed that plants homozygous for mutations in any one of the *TaMs26-a*, *TaMs26-b*, or *TaMs26-d* homeologs were male fertile and capable of generating self-pollinated seed similar to wild-type plants [19]. These results suggested that no single *TaMs26* homeolog was functionally dominant and that a combination of two or all three homeologs contributed towards male fertility. To examine the impact of homozygous mutations in two genomes on male fertility, *TaMs26-a*, *TaMs26-b*, or *TaMs26-d* mutations were combined through conventional crossing. As shown in Table 1, double homozygous mutant pairs were generated in combinations that retained a wild-type genome for *TaMs26-A*, *TaMs26-B* or *TaMs26-D*. These double mutants showed normal vegetative and floral growth and were male fertile, generating viable pollen as indicated by seed set on these plants that was comparable to wild-type plants (Table 1). This data demonstrated that plants wild-type for *TaMs26* in any one genome were competent to maintain male fertility. Moreover, plants that contained a homozygous mutation in one genome and a heterozygous wild-type allele in each of the other two genomes were also male fertile; for example, plants that contained homozygous 4 bp Δ, 81 bp Δ/WT and 90 bp Δ/WT in *TaMs26-A*, *TaMs26-B* and *TaMs26-D*, respectively, were male fertile and set seed similar to wild-type plants. This observation demonstrated that two wild-type *Ms26/CYP704B* alleles derived either from a single genome or from two different genomes were sufficient for male fertility in wheat.

**Table 1:** Male fertility of double and triple *TaMs26* mutants.

Genotype of *TaMs26* homeologs^a^	*TaMs26-A*	*TaMs26-B*	*TaMs26-D*	Male Fertility^b^
*TaMs26*-*Abd*	WT	9 bp Δ	96 bp Δ + 2bp I	Fertile
*TaMs26*-*Abd*	WT	81 bp Δ	96 bp Δ + 2bp I	Fertile
*TaMs26*-*aBd*	4 bp Δ	WT	96 bp Δ + 2bp I	Fertile
*TaMs26*-*abD*	4 bp Δ	81 bp Δ	WT	Fertile
*TaMs26*-*abD*	4 bp Δ	23 bp Δ	WT	Fertile
*TaMs26*-*aBbDd*	4 bp Δ	WT:81 bp Δ	WT:90 bp Δ	Fertile
*TaMs26*-*AabDd*	WT:4 bp Δ	81 bp Δ	WT:90 bp Δ	Fertile
*TaMs26*-*AaBbd*	WT:4 bp Δ	WT:81 bp Δ	90 bp Δ	Fertile

^a^ WT *TaMs26* allele represented by uppercase letter (*A*, *B*, *D*) and *TaMs26* mutant allele represented by lowercase letter (*a*, *b*, *d*).

^b^ Plants with an average seed set >100 were scored as fertile.

### Triple recessive mutations in *TaMs26* result in male sterility

**Fig 3:**
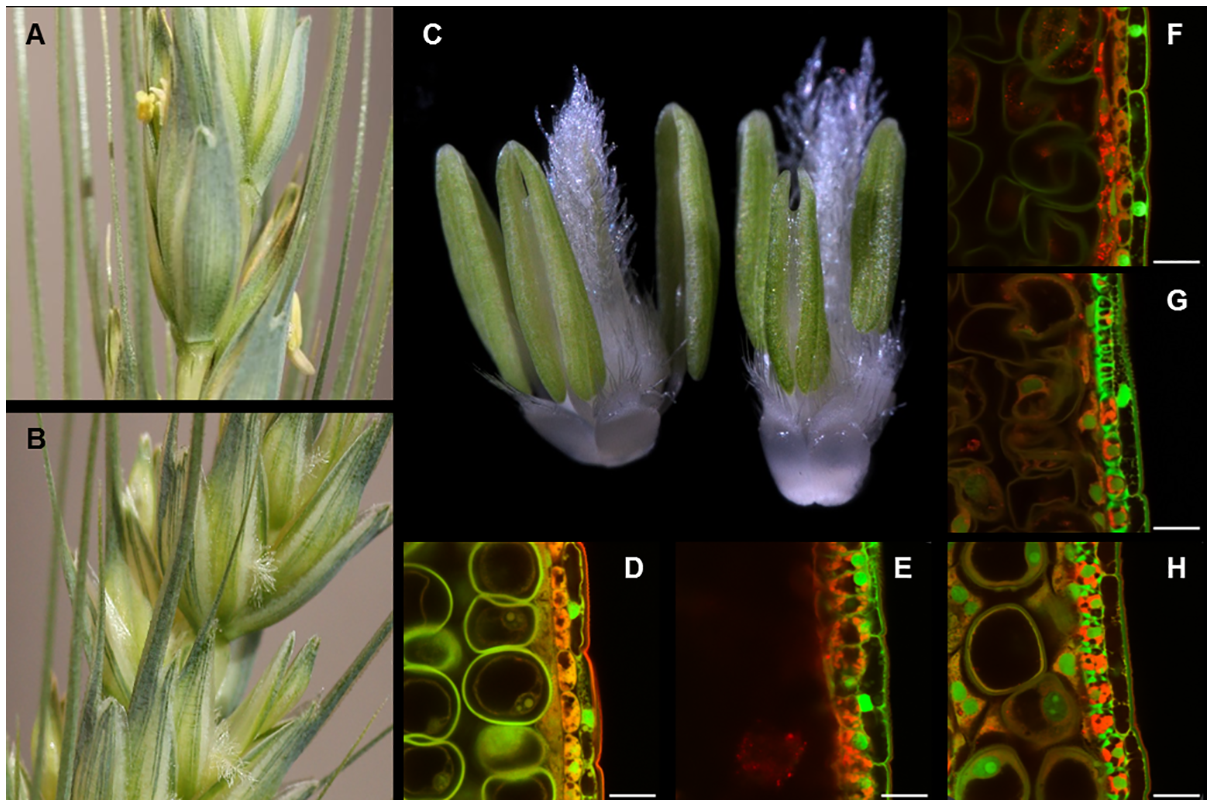
Anther and microspore phenotypes from wild type and *Tams26* mutant wheat plants. Spike from wild type (A) and *Tams26*-*abd* mutant (B) plants. (C) Anthers and ovary from flower of wild type (left) and *Tams26*-*abd* mutant (right) plants. Microspores at late vacuolate stage from wild type, *TaMs26*-*ABD* (D); triple recessive, *Tams26*-abd (E); and double homozygous-single heterozygous: *Tams26*-*Aabd* (F), *Tams26*-*aBbd* (G), and *Tams26*-*abDd* (H) plants. (D) and (E) comparison of sections of anthers from a wild type plant vs a triple homozygous mutant. (F), (G) and (H) illustrate the differences in pollen morphology from double homozygous-single heterozygous mutants which are heterozygous for A-, B- and D-genomes respectively. Scale bars = 25 μm.

**Fig 4:**
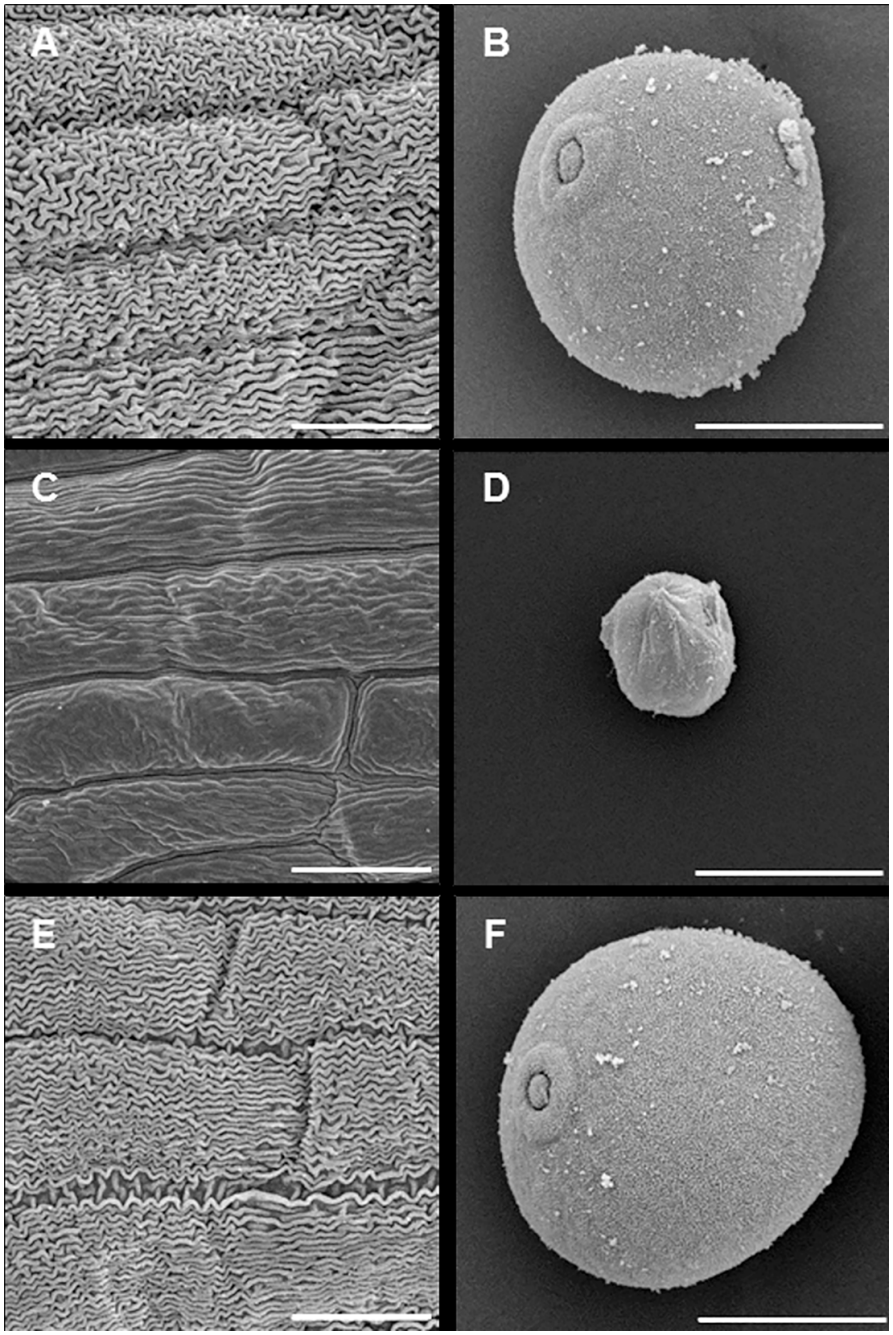
Scanning electron microscopy of anther epidermis and microspores at late vacuolate stage of microspore development. Wild type (A and B), *Tams26-abd* (C and D), and *Tams26-abd*/*ZmMs26*-*OsMs26* (E and F) plants. Scale bars = 20 μm.

Triple homozygous mutants (*Tams26*-*abd*) were generated through conventional crossing to examine male fertility in the absence of a functional copy of *TaMs26*. Individual plants containing double homozygous *TaMs26* mutants were crossed with plants containing a single homozygous *TaMs26* mutation. Utilizing the above mentioned five deletions in A-, B- and D-genomes, three sets of triple mutants were generated i) A-4 bp Δ, B-23 bp Δ, D-90 bp Δ; ii) A-4 bp Δ, B-81 bp Δ, D-90 bp Δ; iii) A-4 bp Δ, B-23 bp Δ, D-96 bp Δ + 2 bp I. Plants heterozygous for triple mutations (*TaMs26-AaBbDd*) were allowed to self-pollinate and progeny plants homozygous, heterozygous, and wild-type for each of the three mutations were identified. These plants were evaluated for vegetative and reproductive phenotypes. All triple homozygous mutants (*Tams26-abd*) were similar to wild-type plants with no obvious differences in vegetative growth characteristics or flowering characteristics (Fig 3A and 3B). Flowers from the *Tams26-abd* plants were identical to flowers from wild-type plants with the exception that anthers from the *Tams26-abd* plants were visibly shriveled in appearance (Fig 3C). The presence of microspores was examined in the triple mutants and wild-type plants through confocal microscopy of anthers at the vacuolate stage of microspore development. In other grasses, such as maize, rice, and sorghum, mutations in the ortholog of the *Ms26/CYP704B* resulted in the breakdown of microspores shortly after tetrad release [15, 18, 19]. As shown in Fig 3D and 3E, anthers from a wild-type plant contained a high density of vacuolate microspores, while anthers from a *Tams26-abd* plant contained only a few vestigial microspores. These vestigial microspores were shrunken and appeared to have a deformed pollen wall compared to pollen from wild-type plants when examined by scanning electron microscopy (SEM) (Fig 4).

As has been shown in rice, *CYP704B2* also has a role in the formation of anther cuticle. Unlike wild-type anthers, *cyp704b2* mutant anthers exhibit abnormal epidermal cuticle formation which is smooth and glossy [15]. To investigate whether *TaMs26* is similarly involved in anther cuticle formation, the anther epidermis at vacuolate microspore stage in the *Tams26* mutants was analyzed by SEM. In contrast to the epidermis of anthers from wild-type plants, which have well-developed cuticular and epicuticular structures (Fig 4A), the epidermal wall of anthers from *Tams26*-*abd* mutants was smooth and devoid of these cuticular structures (Fig 4C). These observations demonstrated that, like rice, *TaMs26* has a central role both in anther epidermis and pollen wall development in wheat.

### One wild-type *TaMs26* allele is insufficient for maintaining male fertility

Interestingly, in addition to the triple homozygous mutants, anther structure and pollen viability was also affected in double homozygous-single heterozygous plants. Despite the presence of a single functional allele, seed number was severely reduced in these plants when compared to wild-type plants (Table 2). Seed set in these mutant plants ranged from 0–32 seeds per plant and was dependent upon which genome contributed the wild-type allele. The lowest seed set was observed in *Tams26*-*aBbd* mutants and highest in *Tams26*-*abDd* mutants (Table 2). These observed differences in genome contribution were more readily apparent when microspore development was analyzed in these plants. The most severe pollen phenotype was observed in *Tams26*-*aBbd* plants, which contained anthers that had severely collapsed and translucent microspores (Fig 3G). The least severe phenotype was observed in anthers from *Tams26*-*abDd* plants, which contained numerous normal-looking microspores and a nearly equal number of collapsed and shrunken microspores (Fig 3H). The pollen phenotype in *Tams26*-*Aabd* plants was intermediate to that of *Tams26*-*aBbd*, and *Tams26*-*abDd* plants (Fig 3F). The cuticle phenotype in the double homozygous-single heterozygous mutant plants also mirrored the observations on microspore phenotype. Anthers in *Tams26*-*aBbd* plants exhibited the most severe defects and were very similar to those from *Tams26*-*abd* plants in smoothness of epidermal surface (S3 Fig) while anthers from *Tams26*-*abDd* plants exhibited the least severe phenotype and closely resembled the wild-type anthers (S3 Fig). The observed differences in anther wall and microspore morphology parallel the seed set across the different single heterozygous *TaMs26* genomes. Together, these results clearly demonstrate that one wild-type *TaMs26* allele is insufficient to maintain male fertility in wheat and the alleles from three genomes are functionally non-equivalent.

**Table 2:** Male fertility of triple *TaMs26* mutants as determined by seed set.

Genotype of *TaMs26* homeologs^a^	Seed Set—Fertility			
	Plants^b^	Total Seed	Seed per Plant	
			Mean	Range
*Tams26*-*abd*	17	4	0.25	0–3
*Tams26*-*Aabd*	37	210	5.7	0–13
*Tams26*-*aBbd*	24	33	1.4	0–5
*Tams26*-*abDd*	19	296	15.6	7–32
*TaMs26-ABD*	3	444	148.0	121–186

^a^ WT *TaMs26* allele represented by uppercase letter (*A*, *B*, *D*) and *TaMs26* mutant allele represented by lowercase letter (*a*, *b*, *d*). Results from three combinations of *TaMs26* mutations in A-, B-, and D-genomes (i) *TaMs26*-*a* 4bp Δ, *TaMs26*-*b* 81 bp Δ, and *TaMs26-d* 90 bp Δ; (ii) *TaMs26*-*a* 4bp Δ, *TaMs26*-*b* 23 bp Δ, and *TaMs26-d* 90 bp Δ; and (iii) *TaMs26*-*a* 4bp Δ, *TaMs26*-*b* 81 bp Δ, and *TaMs26-d* 96 bp Δ + 2 bp I; Δ, deletion, and I, insertion.

^b^ Heads were not bagged. We observed negligible cross pollination in greenhouse conditions; however, residual pollination was possible.

### Complementation of triple homozygous mutants requires two *Ms26/CYP704B* genes

To further ascertain that the male sterility in *Tams26*-*abd* plants was directly due to mutations in *TaMs26* genes, complementation was tested with the maize *Ms26* gene. A copy of the maize *Ms26* gene (also referred to as *ZmMs26*) under control of the native maize *Ms26* promoter was introduced into wild-type wheat plants (cv. Fielder) using *Agrobacterium*-mediated transformation. Two independent single-copy T-DNA insertions were identified by molecular analyses and crossed as females with pollen from wheat plants heterozygous for the *TaMs26*-*A* 4 bp Δ, *TaMs26*-*B* 81 bp Δ, and *TaMs26-D* 96 bp Δ + 2bp I mutant alleles. Progeny of these crosses was screened to confirm the presence of *ZmMs26* and *TaMs26* mutant alleles, and allowed to self-pollinate. F2 seed from this generation were planted and progeny genotyped for the introduced *ZmMs26* gene and *TaMs26* mutant alleles. Seed from these individual plants was counted as a qualitative measure of male fertility. As shown in Tables 2 and 3, in contrast to the low or no seed set observed with double homozygous-single heterozygous mutants, seed set was restored to wild-type levels when these plants contained a transformed copy of *ZmMs26*. However, *ZmMs26* was unable to restore male fertility to *Tams26*-*abd* mutants when present as a single copy, and no seed set was observed from plants containing either of the two independent *ZmMs26* insertions (Table 3).

**Table 3:** Complementation of *TaMs26* triple recessive mutants.

Genotype of *TaMs26* homeologs^a^	Complementing gene(s)^b^^,^^c^	Seed Set—Fertility			Male fertile	Male sterile
		Plants^d^	Total Seed	Seed per Plant		
*Tams26*-*Aabd*	*ZmMs26*	8	503	62.9	8	0
*Tams26*-*aBbd*	*ZmMs26*	5	509	101.8	5	0
*Tams26*-*abDd*	*ZmMs26*	5	540	108.0	5	0
*Tams26*-*abd*	*ZmMs26*	5	0	0.0	0	5
*Tams26*-*abd*	*ZmMs26* + *OsMs26*	18	1721	95.6	18	0
*Tams26*-*abd*	N/A	19	0	0.0	0	19

^a^ WT *TaMs26* allele represented by uppercase letter (*A*, *B*, *D*) and *TaMs26* mutant allele represented by lowercase letter (*a*, *b*, *d*). Results from combination of *TaMs26* mutations in A-, B-, and D-genomes: *TaMs26*-*a* 4bp Δ, *TaMs26*-*b* 81 bp Δ, and *TaMs26-d* 96 bp Δ + 2 bp I; Δ, deletion.

^b^ *ZmMs26*, maize *Ms26*; *OsMs26*, rice *Ms26*; and N/A, not applicable

^c^ *ZmMs26* includes events #1 and #2, *ZmMs26* +*OsMs26* includes events #3 and #4

^d^ Heads were not bagged. We observed negligible cross pollination in greenhouse conditions; however, residual pollination was possible.

When coupled with the observation of male fertility in the double homozygous mutants and the single homozygous-double heterozygous mutants, observations from single gene complementation strongly suggested that two copies of the *Ms26/CYP704B* gene would be required to complement triple homozygous mutants. To test this hypothesis, the rice *CYP704B2* gene (also referred as *OsMs26*) was added to a T-DNA expression cassette containing the *ZmMs26* gene. Two *Ms26* orthologs from maize and rice were selected based on the demonstration of function of these genes in previous studies [15, 19]. This T-DNA expression cassette was transformed into double homozygous mutants (*TaMs26-Abd*), which were then crossed with A-genome homozygous mutants (*TaMs26-aBbDd*) to generate a population of triple homozygous mutants with and without the *ZmMs26*-*OsMs26* expression cassette. In total, 37 triple homozygous *Tams26*-*abd* mutant plants were analyzed, 18 with one copy of *ZmMs26*-*OsMs26* and 19 lacking the T-DNA expression cassette. All 18 *Tams26*-*abd* plants that carried *ZmMs26*-*OsMs26* were male fertile with seed set comparable to wild-type plants and all 19 plants without the T-DNA expression cassette were male sterile (Table 3). It was observed that both the anther epidermis and pollen wall phenotypes were restored to the wild-type phenotype in the plants carrying the *ZmMs26*-*OsMs26* expression cassette (Fig 4E and 4F). Taken together, these results indicated that two functional alleles of *Ms26/CYP704B* gene are required for anther and pollen development in wheat.

## Discussion

### *Ms26/CYP704B* genes show high conservation of structure and function in wheat and other grasses

Sporopollenin, the main constituent of pollen exine is highly conserved both in structure and composition. Orthologs of the sporopollenin biosynthesis pathway, including *Acos5*, *PksA*, *PksB*, *CYP703A* and *CYP704B*, are conserved among flowering plants but are not present in green algae [10]. Several studies for expression and functional analysis of these genes support their conserved role. The *Physcomitrella patens PpASCL* is a functional ortholog of *Arabidopsis PKSA* as it is capable of hydroxyalkylpyrone synthase activity with *in vitro* preference for hydroxyl fatty acyl-CoA esters [26]. The expression profile of the *CYP704B* gene family in plant species from bryophytes to angiosperms is conserved to inflorescences, male organs, and spores highlighting their evolutionary protective role in spores [15, 27, 28]. Similar to *Arabidopsis*, rice and maize *Ms26*/*CYP704B* orthologs that exhibit male reproductive tissue preferential expression [14–16], the expression pattern of *TaMs26* homeologs is anther-specific. Furthermore, all three *TaMs26* homeologs were observed to be expressed at comparable levels implying that each allele contributes to CYP704B function during pollen development in wheat. The role of *MS26/CYP704B* orthologs in pollen exine formation has also been functionally demonstrated across plants. *Arabidopsis*, rice, and maize mutants of *Ms26/CYP704B* orthologs have been shown to have defects in pollen exine structure [14, 15, 18]. Similarly, the triple homozygous wheat (*TaMs26-abd*) mutants were male sterile due to defects in microspore morphology.

Interestingly, compared to wheat and other grass species, the *Arabidopsis cyp704B1* mutants exhibit a milder phenotype. Pollen in *cyp704B1* mutant plants lack a normal exine but remain viable and can set seed upon self-pollination [14]; whereas *Ms26/CYP704B* mutants in wheat, rice, maize, and sorghum are male sterile. In addition to microspore exine defects, wheat *Tams26* and rice *cyp704B2* mutants also exhibit defects in anther epidermis [15]. Although it is not known if anther epidermis is similarly affected in *Arabidopsis cyp704B1* mutants, it is possible that the male sterility in wheat and rice (and possibly maize and sorghum) is due to a combination of anther epidermis and pollen exine defects. If so, then the *CYP704B2* genes in wheat and rice (and other grasses) could have assumed a more central role by contributing not only to pollen exine formation but also to anther epidermis formation. In *Arabidopsis*, other members of the CYP86 clan such as those involved in cutin formation [29, 30] may contribute towards anther epidermis development.

### All three *TaMs26* homeologs contribute towards male fertility but with underlying differences in contribution

Bread wheat is an allohexaploid that originated from two hybridization events involving three progenitor species [20, 21]. Such inter-specific hybridization events are generally accompanied by genetic or epigenetic resetting to orchestrate gene expression from the complex allopolyploid genome [31]. Resetting can result in genomic asymmetry or expression of individual genes from specific genomes and can lead to partial or complete genome dominance (reviewed in Madlung and Wendel [32]). In wheat, such partial or complete genome dominance is evident for several traits including free threshing phenotype and brittle rachis which are related to crop domestication [20]. Analysis of *TaMs26* double homozygous and triple homozygous mutants suggested functional equivalency of all three *TaMs26* homeologs. *Ms26/CYP704B* genes have also been analyzed in *Brassica napus*, a tetraploid species, and it was observed that a double knockout in duplicate functionally redundant genes, *BnMs1* (*BnCYP704B1-1*) and *BnMs2* (*BnCYP704B1-1*), is required to obtain complete loss of function, which results in male sterility. However, in wheat, the analysis of the double homozygous-single heterozygous mutants revealed noticeable differences in the contribution of each genome. Pollen and anther phenotypes, and consequently seed set, of these mutants were dependent on the genome that contributed the heterozygous allele. The fertility of double homozygous-single heterozygous mutants was highest when the heterozygous allele was contributed by D-genome (*Tams26*-*abDd*). These observations suggested that the function of *Ms26/CYP704B* in *Tams26*-*abDd* plants was likely reduced but still significant. On the other hand, anther and pollen phenotypes as well as seed set were lowest when the heterozygous allele was contributed by the B-genome (*Tams26*-*aBbd*). When analyzed as a single wild-type allele in a background of five mutant alleles there are noticeable functional differences between the three *TaMs26* homeologs. These differences suggest dominance, although subtle, of the D-genome over A- and B-genomes for this gene. In contrast, it was observed in *Brassica napus* that the genotypes *BnMs1ms1ms2ms2* and *Bnms1ms1Ms2ms2* exhibit wild-type male fertility phenotype [33, 34]. Transcript counts for the three wheat homeologs in anthers showed comparable levels, suggesting that the differences observed in phenotype were not due to differential transcription of the homeologs. These can likely be attributed to post-transcriptional regulation. Differences in protein sequences can also lead variable enzymatic activity and although the predicted amino acid sequences of the three homeologs show 98–99% amino acid identity, several differences in amino acid sequence exist that can have a role in protein function.

### Two functional alleles of *CYP704B/Ms26* are required for male fertility in wheat

Analyses of double and triple homozygous *TaMs26* mutants suggested that two wild-type *TaMs26* alleles are absolutely required for male fertility. These two wild-type alleles can be contributed by a single genome or two different genomes. The complementation experiments with one and two heterologous copies of *Ms26*/*CYP704B* genes further augmented this conclusion. When one copy of *ZmMs26* gene was introduced into a background of triple homozygous mutants (*Tams26-abd*), the heterologous gene was unable to restore male fertility. However, when one copy of *ZmMs26* gene was introduced into a background of double homozygous-single heterozygous mutants, male fertility was completely restored irrespective of which genome contributed the heterozygous alleles. This observation was further borne out by the requirement of two exogenously supplied *Ms26* genes, *ZmMs26* and *OsMs26*, to restore fertility to *Tams26-abd* mutant plants. Under the conditions tested in this report, it is evident that two functional alleles of an *Ms26*/*CYP704B* gene are required for male fertility in wheat whether derived from two endogenous wild-type alleles or orthologous genes from different species.

Previous studies have shown that a single copy of *ZmMs26* gene can complement *ms26/ms26* mutants in maize and sorghum, both diploid species [16, 19]. While a single wild-type allele of *Ms26*/*CYP704B* gene is sufficient for normal function in diploid species, data presented here indicates that hexaploid wheat has evolved to require at least two functional alleles. It appears that due to the presence of six functional alleles, the polyploid wheat has evolved to require a higher dosage of MS26/CYP704B as compared to diploid species. Interestingly, in the case of *Ms1* gene, another male fertility gene in wheat, the existing data suggests that only the B-genome homeolog has functional relevance. In certain genetic backgrounds, homozygous recessive mutations in the B-genome homeolog resulted in complete male sterility [35]. While *Ms1* functions as a gene in diploid species and heterozygotes (*Ms1/ms1*) are male fertile, *TaMs26* likely represents the other end of the spectrum where all three genomes contribute towards a phenotype, but the minimum requirement for male fertility is two wild-type alleles.

## Conclusion

Knowledge of genes contributing to reproductive development in wheat has lagged other plant species as polyploidy in wheat makes it less amenable for functional genomics using traditional approaches. However, the development of new biological tools has facilitated the genesis of functional analysis of genes in wheat [36, 37]. In addition to new advances in DNA sequencing, DNA double-strand break (DSB) technologies have provided efficient methods to obtain multiple mutant alleles in the different wheat genomes. Using a custom designed homing endonuclease, mutations were previously generated in each of the three homeologs of the wheat *TaMs2*6 gene [19]. These mutants allowed for the characterization of their gene products to further the understanding of anther and pollen development in wheat. Moreover, the generation of mutant alleles and the ability to restore fertility using orthologous *Ms26* genes sets the foundation for the development of a *TaMs26*-based male fertility control system for hybrid seed production and exploitation of heterosis to increase wheat yields.

## Materials and methods

### Plant material and growth conditions

The *TaMs26* mutant stocks were derived from the single-genome mutations generated by Cigan et al. [19]. Plants were grown in the greenhouse at DuPont Pioneer research facilities in Johnston, Iowa. The greenhouse conditions were set to 22°C and 20°C average day and night temperatures, respectively, with 30% humidity and 16 hr day length period. Standard plant care practices were followed including fertilizer and pesticide applications.

### DNA and protein sequence analysis of *TaMs26* homeologs

To obtain complete genomic sequence for wheat A-, B- and D-homeologs of *Ms26/CYP704B*, TGACv1 (The Genome Analysis Centre) sequence database was BLAST (Basic Local Alignment Research Tool; Altschul et al. [25]) searched with the maize *Ms26* mRNA sequence (accession NM_001137176). *TaMs26* sequences were annotated for exons, introns, and CDS and translated into predicted amino acids based on maize sequence. Sequence comparisons and alignments were performed with DNA analysis software: Sequencher (Genecodes) and Vector NTI (Life Technologies Inc.). Sequences of sorghum (XP_002465796), rice (XP_015629295.1), barley (BAK08270), and *Brachypodium* (XP_003558727.1) orthologs of maize *Ms26* were obtained from the NCBI database.

### Genome-specific PCR assay and sequence analysis

For amplification of *TaMs26* genomic fragments, DNA from *T*. *aestivum* (cv. Fielder) was extracted from four small leaf punches (0.5 cm in diameter) as described in Gao et al. [38]. PCR was performed using Phusion^®^ High Fidelity PCR Master Mix (New England Biolabs Inc.) according to the manufacturer’s recommendations. PCR amplified fragments were also subcloned for DNA sequence analysis using the pCR2.1-TOPO cloning vector (Life Technologies Inc.). A genomic fragment corresponding to *TaMs26* Exon 4 was amplified using primer pair UNIMS26 5’-2 and UNIMS26 3’-1 (S1 Table), which are conserved across the three genomes. Genome-specific fragments were amplified using one genome-specific primer and a second UNIMS primer. A-genome fragment was amplified with primers *TAMS26-A* and UNIMS26 3’-1; B-genome fragment was amplified with primers *TAMS26-B* and UNIMS26 5’-2; and D-genome fragment was amplified with primers *TAMS26*-*D* and UNIMS26 5’-2 (S1 Table). For genotyping larger deletions (*TaMs26-B* 81 bp Δ, *TaMs26-D* 90 bp Δ, and *TaMs26-D* 96 bp Δ+ 2 bp I), corresponding fragments were amplified with genome-specific primers and size fractionated on 2% agarose gel. Smaller deletions (*TaMs26-A* 4 bp Δ and *TaMs26-B* 23 bp Δ) were amplified with corresponding genome-specific primers and part of the amplification product digested with *Bsi*WI, the DNA restriction enzyme that recognizes the sequence 5’-CGTACG-3’, as described in Djukanovic et al. [17]. Products of these reactions were electrophoresed on 1% agarose gels and screened for *Bsi*WI digestion resistant PCR products indicative of mutations at the *Ms26* target site.

### RT-PCR analysis and transcript counts through deep sequencing

Anthers were collected for stages corresponding to pre-meiotic stage, meiosis II (tetrad) stage, early uninucleate microspores, and late uninucleate microspores. Ovaries were collected at stages corresponding to meiosis and gametogenesis. Leaf and root tissue were collected at seedling stage. RNA was extracted using TRIZOL reagent (Invitrogen Inc.) following manufacturer’s protocol. A total of 1 mg RNA was treated with DNase I enzyme (Invitrogen Inc.) following the manufacturer’s recommendations to remove DNA contamination. 500 ng of DNase-treated RNA was reverse transcribed with the iScript cDNA synthesis kit (Bio-Rad). The transcribed cDNA was diluted 5X in sterile water and used for PCR. Primer pair *TAMS26*-F and *TAMS26*-R was used to amplify *TaMs26* transcript and primer pair *TAACTIN*-F and *TAACTIN*-R was used to amplify *TaActin*, and products were analyzed by gel electrophoresis. qRT-PCR was performed with PowerUp SYBR Green Master Mix (ThermoFisher Scientific) with above mentioned primer pairs on a ViiA^™^7 System (ThermoFisher Scientific). Relative quantification values were determined using the difference in Ct from the *TaMs26* and the reference gene (*TaActin*).

For transcript count by deep sequencing, RT-PCR was performed on the cDNA synthesized above with Phusion^®^ High Fidelity PCR Master Mix (New England Biolabs) adding on the sequences necessary for amplicon-specific barcodes and Illumina sequencing using “tailed” primers through two rounds of PCR. The primers used in the primary PCR reaction were *Ms26*F and *Ms26*R while the primers used in the secondary PCR reaction were F2 and R2. The product amplified by these primers contained two single nucleotide polymorphisms (SNPs) that could discriminate between the three homeologs. The resulting PCR products were concentrated using a Qiagen MinElute^®^ PCR purification spin column (QIAGEN, Inc.). A portion of the amplification products was visualized on an agarose gel and the remainder was single read 100 nt-length deep sequenced on an Illumina Genome Analyzer IIx (ELIM Biopharmaceuticals, Inc.) with a 30–40% (v/v) spike of PhiX control v3 (Illumina, Inc.) to off-set sequence bias. Two independent reactions were performed. The reads were clustered based on their SNPs and the number of reads was counted using in-house designed sequence analysis software.

### Ms26 complementation constructs and transformation

To generate the *ms26* fertility complementation T-DNA vector, the *Ms45* gene in PHP24490 [39] was replaced by a 3.9 kb DNA fragment [Chromosome 1, nt 4509689–14506399 Maize (B73) RefGen_v2 (MGSC)], which contained the wild-type maize *Ms26* gene (also referred to as *ZmMs26*). The T-DNA expression cassette also carried an *α-amylase* gene under the control of pollen-specific PG47 promoter along with *DsRed* gene regulated by a seed-specific promoter as the transformation selectable marker. The α-amylase prevented the pollen-specific transmission of the *ZmMs26* gene and resulted in 50:50 segregation of *ZmMs26* in transformed plants. The final T-DNA expression cassette, pPG47::Bt1:ZmAA1//Ms26//35SEN-pEND2::DsRed2, was introduced into *Agrobacterium* strain LBA4404 as described above. To make the construct with two *Ms26* orthologous genes, a 3.2.0 kb genomic fragment of the rice *Ms26*/*CYP704B* gene (LOC_Os03g07250.1; chromosome 3 nt 3701612–3704874, Os-Nipponbare ref IRGSP-1.0MSU Rice Genome Annotation Project) including approximately 1.0 kb fragment 5’ of the ATG translation start site and 0.5 kb 3’ of the translation stop site, was amplified from *Orzya sativa* L. ssp. Japonica, cv. Kitaake, with primers *OSMS26*-F and *OSMS26*-R. Amplification and sequencing was performed as described for the wheat *TaMs26* genomic fragments. The rice *Ms26* ortholog (also referred to as *OsMs26*) was then cloned into the above mentioned vector, which contained the *ZmMs26* gene. The T-DNA expression cassette was transformed into *T*. *aestivum* (cv. Fielder) containing homozygous *Tams26- a* and *b* mutations as described in Cigan et al. [19]. Transformed plants were crossed with pollen from *Tams26aBbd* plants and progeny analyzed (see S4 Fig for crossing strategy). Plants transformed with *ZmMs26* and *ZmMs26-OsMs26* constructs were genotyped with the *ZMMS26 TERM* primer pair and *OSMS26PRO* primer pair, respectively (S1 Table).

### Microscopy

For scanning electron microscopy (SEM), anthers and pollen were fixed in FAA (formalin, acetic acid, EtOH) and dehydrated in a graded EtOH series (50%, 75%, 95%, 100%). At this point, the samples were critical point dried using liquid carbon dioxide (Denton Vacuum). Anthers and pollen were mounted on aluminum stubs with double-sided sticky tabs, and surrounded with silver paint. The samples were then sputter-coated (Denton Vacuum) with palladium-gold (60:40) and observed using a JEOL 5800 scanning electron microscope (JEOL USA, Inc.) at 10 kV.

For confocal scanning laser microscopy (CSLM), flowers were collected and fixed in 2% paraformaldedye: 4% glutaraldehyde in PBS buffer and vacuum infiltrated at 10 psi to increase the penetration of fixative. Samples were rinsed in PBS and then cleared in graded series of 2,2’-Thiodiethanol (TDE) (10%, 25%, 50%, 75%, and 97%). Samples were mounted on slides in TDE and imaged using the Leica TCS SPE CSLM (Lecia Microsystems) with 405 nm, 488 nm and 532 nm laser lines. 20 anthers from each mutant class and wild type plants were analyzed.

## Supporting information

S1 Fig Comparison of genomic sequence of *TaMs26* homeologs of wheat. *TaMs26* gene sequences corresponding to A-, B-, and D-genomes are shown with annotations of exons and introns.
(PDF)

S2 Fig Spatial and temporal expression of *TaMs26* determined by semi-quantitative RT-PCR. *TaActin* expression was used as a control. P, pre-meiosis; M, meiosis II; E, early uninucleate; L, late uninucleate; G, gametogenesis; gDNA, genomic DNA.
(PDF)

S3 Fig Scanning electron microscopy of anther epidermis from double homozygous-single heterozygous *Tams26* mutant wheat plants. Anther epidermis surface at late vacuolate microspore stage from wild type (A), *Tams26-Aabd* (B), *Tams26-abDd* (C), and *Tams26-aBbd* (D) plants. Scale bars = 20 μm.
(PDF)

S4 Fig Crossing strategy to combine wheat mutations with transformed maize and rice genes for complementation testing. (PDF)

S1 Table List of primers used in the study. (PDF)
