# Supplementary material for: MS26/CYP704B is required for anther and pollen wall development in bread wheat (Triticum aestivum L.) and combining mutations in all three homeologs causes male sterility
Source: PLoS One. 2017 May 16;12(5):e0177632. doi: 10.1371/journal.pone.0177632 (PMC5433722; doi:10.1371/journal.pone.0177632)
Supplement: S1 Fig — TaMs26 gene sequences corresponding to A-, B-, and D-genomes are shown with annotations of exons and introns. (PDF) [file pone.0177632.s001.pdf]

Supporting Information

```
          1                                                 50
TaMS26-A  ATGAGCAGCC CCATGGAGGA AGCTCACCAT GGCATGCCGT CGACGACGAC
TaMS26-B  ATGAGCAGCC CCATGGAGGA AGCTCACCTT GGCATGCCGT CGACGACG--
TaMS26-D  ATGAGCAGCC CCATGGAGGA AGCTCACGGC GGCATGCCGT CGACGACG--

          51                                               100
TaMS26-A  GGCGTTCTTC CCGCTGGCAG GGCTCCACAA GTTCATGGCC ATCTTCCTCG
TaMS26-B  -GCCTTCTTC CCGCTGGCAG GGCTCCACAA GTTCATGGCC GTCTTCCTCG
TaMS26-D  -GCCTTCTTC CCGCTGGCAG GGCTCCACAA GTTCATGGCC ATCTTCCTCG

          101                                              150
TaMS26-A  TGTTCCTCTC GTGGATCTTG GTCCACTGGT GGAGCCTGAG GAAGCAGAAG
TaMS26-B  TGTTCCTCTC GTGGATCCTG GTCCACTGGT GGAGCCTGAG GAAGCAGAAG
TaMS26-D  TGTTCCTCTC GTGGATCTTG GTCCACTGGT GGAGCCTGAG GAAGCAGAAG

          151                                              200
TaMS26-A  GGGCCGAGGT CATGGCCGGT CATCGGCGCG ACGCTGGAGC AGCTGAGGAA
TaMS26-B  GGGCCACGGT CATGGCCGGT CATCGGCGCG ACGCTGGAGC AGCTGAGGAA
TaMS26-D  GGGCCGAGGT CATGGCCGGT CATCGGCGCG ACGCTGGAGC AGCTGAGGAA

          201                                              250
TaMS26-A  CTACTACCGG ATGCACGACT GGCTCGTGGA GTACCTGTCC AAGCACCGGA
TaMS26-B  CTACTACCGG ATGCACGACT GGCTCGTGGA GTACCTGTCC AAGCACCGGA
TaMS26-D  CTACTACCGG ATGCACGACT GGCTCGTGGA GTACCTGTCC AAGCACCGGA

          251                                              300
TaMS26-A  CGGTCACCGT CGACATGCCC TTCACCTCCT ACACCTACAT CGCCGACCCG
TaMS26-B  CGGTCACCGT CGACATGCCC TTCACCTCCT ACACCTACAT CGCCGACCCG
TaMS26-D  CGGTGACCGT CGACATGCCC TTCACCTCCT ACACCTACAT CGCCGACCCG

          301                                              350
TaMS26-A  GTGAACGTCG AGCATGTGCT CAAGACCAAC TTCAACAATT ACCCCAAGGT
TaMS26-B  GTGAACGTCG AGCACGTGCT CAAGACCAAC TTCAACAATT ACCCCAAGGT
TaMS26-D  GTGAACGTCG AGCATGTGCT CAAGACCAAC TTCAACAATT ACCCCAAGGT

          351                                              400
TaMS26-A  GAAACTGAAA GAACCCCTCA GCCTT--GTG AATTTTTTTG CCAA--GGTT
TaMS26-B  GAAAC----- -AATCCTCGA GATGTCAGTC AAGGTTCAGT ATAATCGGTA
TaMS26-D  GAAAC----- -AATCCTCGA GATGTCAGTA AAGGTTCAGT ATAATCGGTA
                                   Intron
          401                                              450
TaMS26-A  CAGA-AGT-T TACAC-TGAC ACAAATGTCT GAAATTGTAC GTGTAGGGGG
TaMS26-B  CTGACAGTGT TACAAATGTC TGAAAT--CT GGAATTGTGT GTGTAGGGGG
TaMS26-D  CTGACAGTGT TACAAATGTC TGAAAT--CT GAAATTGTAT GTGTAGGGGG

          451                                              500
TaMS26-A  AGGTGTACAG GTCCTACATG GACGTGCTGC TCGGCGACGG CATCTTCAAC
TaMS26-B  AGGTGTACAG GTCCTACATG GACGTGCTGC TCGGCGACGG CATATTCAAC
TaMS26-D  AGGTGTACAG GTCCTACATG GACGTGCTGC TCGGCGACGG CATATTCAAC

          501                                              550
TaMS26-A  GCCGACGGCG AGCTCTGGAG GAAGCAGAGG AAGACGGCGA GCTTCGAGTT
TaMS26-B  GCCGACGGCG AGCTCTGGAG GAAGCAGAGG AAGACGGCGA GCTTCGAGTT
TaMS26-D  GCCGACGGCG AGCTCTGGAG GAAGCAGAGG AAGACGGCGA GCTTCGAGTT

          551                                              600
TaMS26-A  CGCTTCCAAG AACCTGAGAG ACTTTAGCAC GATCGTGTTC AGGGAGTACT
TaMS26-B  CGCTTCCAAG AACTTGAGAG ACTTCAGCAC GATCGTGTTC AGGGAGTACT
TaMS26-D  CGCTTCCAAG AACTTGAGAG ACTTCAGCAC GATCGTGTTC AGGGAGTACT

          601                                              650
TaMS26-A  CCCTGAAGCT GCGCAGCATC CTGAGCCAGG CTTGCAAGGC CGGCAAAGTC
TaMS26-B  CCCTGAAGCT GTCCAGCATC CTGAGCCAGG CTTGCAAGGC AGGCAAAGTT
TaMS26-D  CCCTGAAGCT GTCCAGCATA CTGAGCCAGG CTTGCAAGGC CGGCAAAGTT
```

```
           651                                                700
TaMS26-A   GTGGACATGC AGGTAACCGA ACTCAGTCCC TTGGTCATCT GAACATTGAT
TaMS26-B   GTGGACATGC AGGTAACTGA ACTCTTTCCC TTGGTCATAT GAACGTTGAT
TaMS26-D   GTGGACATGC AGGTAACTGA ACTCATTCCC TTGGTCATCT GAACGTTGAT

           701                                                750
TaMS26-A   TTCTTGGACA AAATTTCAAG ATTCTGACGC GAGCGAGCGA ATTCAGGAGC
TaMS26-B   TTCTTGGACA AAATCTCAAG ATTCTGACGC GAGCGAGCCA ATTCAGGAGC
TaMS26-D   TTCTTGGACA AAATTTCAAG ATTCTGACGC GAGCGAGCGA ATTCAGGAGC
                                    Intron
           751                                                800
TaMS26-A   TGTACATGAG GATGACGCTG GACTCGATCT GCAAGGTGGG GTTCGGGGTC
TaMS26-B   TGTACATGAG GATGACGCTG GACTCGATCT GCAAGGTGGG GTTCGGGGTG
TaMS26-D   TGTATATGAG GATGACGCTG GACTCGATCT GCAAAGTGGG GTTCGGAGTC

           801                                                850
TaMS26-A   GAGATCGGCA CGCTGTCGCC GGAGCTGCCG GAGAACAGCT TCGCGCAGGC
TaMS26-B   GAGATCGGCA CGCTGTCGCC GGAGCTGCCG GAGAACAGCT TCGCGCAGGC
TaMS26-D   GAGATCGGCA CGCTGTCGCC GGAGCTGCCG GAGAACAGCT TCGCGCAGGC

           851                                                900
TaMS26-A   GTTCGACGCC GCCAACATCA TCGTGACGCT GCGGTTCATC GACCCGCTGT
TaMS26-B   CTTCGACGCC GCCAACATCA TCGTGACGCT GCGGTTCATC GACCCGCTGT
TaMS26-D   GTTCGACGCC GCCAACATCA TCGTGACGCT GCGGTTCATC GACCCGCTGT

           901                                                950
TaMS26-A   GGCGCGTGAA GAAGTTCCTG CACGTCGGCT CGGAGGCGCT GCTGGAGCAG
TaMS26-B   GGCGCGTGAA GAAATTCCTG CACGTCGGCT CGGAGGCGCT GCTGGAGCAG
TaMS26-D   GGCGCGTGAA GAAGTTCCTG CACGTCGGCT CGGAGGCGCT GCTGGAGCAG

           951                                               1000
TaMS26-A   AGCATCAAGC TCGTCGACGA GTTCACCTAC AGCGTCATCC GCCGGCGCAA
TaMS26-B   AGCATCAAGC TCGTCGACGA GTTCACCTAC AGCGTCATCC GCCGGCGCAA
TaMS26-D   AGCATCAAGC TCGTCGACGA GTTCACCTAC AGCGTCATCC GCCGGCGCAA

           1001                                              1050
TaMS26-A   GGCCGAGATC GTGCAGGCCC GGGCCAGCGG CAAGCAGGAG AAGGTGCGTA
TaMS26-B   GGCCGAGATC GTGCAAGCCC GGGCCAGCGG CAAGCAGGAG AAGGTGCGTA
TaMS26-D   GGCCGAGATC GTGCAGGCCC GGGCCAGCGG CAAGCAGGAG AAGGTGCGTG

           1051                                              1100
TaMS26-A   CGTGATCGTC GTC----GTC AAGCTCCGGA TCGCTGGTTT GTGTAGGTGC
TaMS26-B   CGTGGTCATC GTCATTCGTC AAGCTCCCGA TCGCTGGTTT GTGCAGATGC
TaMS26-D   CGTGGTCATC GTCATTCGTC AAGCTCCCGG TCGCTGGTTT GTGTAGATGC
                                            Intron
           1101                                              1150
TaMS26-A   CATTGATCAC TGACACACTA GCTGGGTGCG CAGATCAAGC ACGACATACT
TaMS26-B   CACTGATCAC TGACACATTA ACTGGGCGCG CAGATCAAGC ACGACATACT
TaMS26-D   CATGGATCAC TGACACACTA ACTGGGCGCG CAGATCAAGC ACGACATACT

           1151                                              1200
TaMS26-A   GTCGCGGTTC ATCGAGCTGG GCGAGGCCGG CGGGGACGAC GGCGGCAGCC
TaMS26-B   GTCGCGGTTC ATCGAGCTGG GCGAGGCCGG CGGCGACGAC GGCGGCAGCC
TaMS26-D   GTCGCGGTTC ATCGAGCTGG GCGAGGCCGG CGGCGACGAC GGCGGCAGTC

           1201                                              1250
TaMS26-A   TGTTCGGGGA CGACAAGGGC CTCCGCGACG TGGTGCTCAA CTTCGTGATC
TaMS26-B   TGTTCGGGGA CGACAAGGGC CTCCGCGACG TGGTGCTCAA CTTCGTCATC
TaMS26-D   TGTTCGGGGA CGACAAGGGC CTCCGCGACG TGGTGCTCAA CTTCGTGATC

           1251                                              1300
TaMS26-A   GCCGGGCGGG ACACCACGGC CACGACGCTC TCCTGGTTCA CCTACATGGC
TaMS26-B   GCCGGGCGGG ACACGACGGC CACGACGCTC TCCTGGTTCA CCTACATGGC
TaMS26-D   GCCGGGCGGG ACACCACGGC CACGACGCTG TCCTGGTTCA CCTACATGGC

           1301                                              1350
TaMS26-A   CATGACGCAC CCGGCCGTGG CCGAGAAGCT CCGCCGCGAG CTGGCCGCCT
TaMS26-B   CATGACGCAC CCGGCCGTGG CCGAGAAGCT CCGCCGCGAG CTGGCCGCCT
TaMS26-D   CATGACGCAC CCGGACGTGG CCGAGAAGCT CCGCCGCGAG CTGGCCGCCT
```

```
          1351                                                  1400
TaMS26-A  TCGAGGCGGA CCGCGCCCGC GAGGATGGCG TCGCGCTGGT CCCCTGCAGC
TaMS26-B  TCGAGTCCGA GCGCGCCCGC GAGGATGGCG TCGCTCTGGT CCCCTGCAGC
TaMS26-D  TCGAGGCGGA GCGCGCCCGC GAGGATGGCG TCGCTCTGGT CCCCTGCGGC
          1401                                              1450
TaMS26-A  GACTCAGACG GCGACGGCTC CGACGAGGCC TTCGCCGCCC GCGTGGCGCA
TaMS26-B  GAC------G GCGAGGGCTC CGACGAGGCC TTCGCCGCCC GCGTGGCGCA
TaMS26-D  GAC------G GCGAGGGCTC CGACGAGGCC TTCGCTGCCC GCGTGGCGCA

          1451                                              1500
TaMS26-A  GTTCGCGGGG CTGCTGAGCT ACGACGGGCT CGGGAAGCTG GTGTACCTCC
TaMS26-B  GTTCGCGGGA CTCCTGAGCT ACGACGGGCT CGGGAAGCTG GTGTACCTCC
TaMS26-D  GTTCGCGGGG TTCCTGAGCT ACGACGGCCT CGGGAAGCTG GTGTACCTCC

          1501                                              1550
TaMS26-A  ACGCGTGCGT GACGGAGACG CTGCGCCTGT ACCCGGCGGT GCCGCAGGAC
TaMS26-B  ACGCGTGCGT GACGGAGACG CTCCGCCTGT ACCCGGCGGT GCCGCAGGAC
TaMS26-D  ACGCGTGCGT GACGGAGACG CTGCGCCTGT ACCCGGCGGT GCCGCAGGAC

          1551                                              1600
TaMS26-A  CCCAAGGGCA TCGCGGAGGA CGACGTGCTC CCGGACGGCA CCAAGGTGCG
TaMS26-B  CCCAAGGGCA TCGCGGAGGA CGACGTGCTC CCGGACGGCA CCAAGGTGCG
TaMS26-D  CCCAAGGGCA TCGCGGAGGA CGACGTGCTC CCGGACGGCA CCAAGGTGCG

          1601                                              1650
TaMS26-A  CGCCGGCGGG ATGGTGACGT ACGTGCCCTA CTCCATGGGG CGGATGGAGT
TaMS26-B  CGCCGGCGGG ATGGTGACGT ACGTGCCCTA CTCCATGGGG CGGATGGAGT
TaMS26-D  CGCCGGCGGG ATGGTGACGT ACGTGCCCTA CTCCATGGGG CGGATGGAGT

          1651                                              1700
TaMS26-A  ACAACTGGGG CCCCGACGCC GCCAGCTTCC GGCCGGAGCG GTGGATCGGC
TaMS26-B  ACAACTGGGG CCCCGACGCC GCCAGCTTCC GGCCAGAGCG GTGGATCGGC
TaMS26-D  ACAACTGGGG CCCCGACGCC GCCAGCTTCC GGCCGGAGCG GTGGATCGGC

          1701                                              1750
TaMS26-A  GACGACGGCG CGTTCCGCAA CGCGTCGCCG TTCAAGTTCA CGGCGTTCCA
TaMS26-B  GACGACGGCG CCTTCCGCAA CGCGTCGCCG TTCAAGTTCA CGGCGTTCCA
TaMS26-D  GACGACGGCG CCTTCCGCAA CGCGTCGCCG TTCAAGTTCA CGGCGTTCCA

          1751                                              1800
TaMS26-A  GGCGGGGCCG CGGATCTGCC TCGGCAAGGA CTCGGCGTAC CTGCAGATGA
TaMS26-B  GGCGGGGCCG CGGATCTGCC TGGGCAAGGA CTCGGCGTAC CTGCAGATGA
TaMS26-D  GGCGGGGCCG CGGATTTGCC TCGGCAAGGA CTCGGCGTAC CTGCAGATGA

          1801                                              1850
TaMS26-A  AGATGGCGCT GGCCATCCTG TGCAGGTTCT TCAGGTTCGA GCTCGTGGAG
TaMS26-B  AGATGGCGCT GGCCATCCTG TGCAGGTTCT TCAGGTTCGA GCTCGTGGAG
TaMS26-D  AGATGGCGCT GGCAATCCTG TGCAGGTTCT TCAGGTTCGA GCTCGTGGAG

          1851                                              1900
TaMS26-A  GGCCACCCCG TCAAGTACCG CATGATGACC ATCCTCTCCA TGGCGCACGG
TaMS26-B  GGCCACCCCG TCAAGTACCG CATGATGACC ATCCTCTCCA TGGCGCACGG
TaMS26-D  GGCCACCCCG TCAAGTACCG CATGATGACC ATCCTCTCCA TGGCGCACGG

          1901                                  1937
TaMS26-A  CCTCAAGGTC CGCGTCTCCA GGGCGCCGCT CGCCTGA
TaMS26-B  CCTCAAGGTC CGCGTCTCCA GGGTGCCGCT CGCCTGA
TaMS26-D  CCTCAAGGTC CGCGTCTCCA GGGCGCCGCT CGCCTGA
```

S1 Fig. Comparison of genomic sequence of *TaMs26* homeologs of wheat. *TaMs26* gene sequences corresponding to A-, B- and D-genomes are shown with annotations of exons and introns (shaded).
