# Supplementary material for: MS26/CYP704B is required for anther and pollen wall development in bread wheat (Triticum aestivum L.) and combining mutations in all three homeologs causes male sterility
Source: PLoS One. 2017 May 16;12(5):e0177632. doi: 10.1371/journal.pone.0177632 (PMC5433722; doi:10.1371/journal.pone.0177632)
Supplement: S2 Fig — TaActin expression was used as a control. P, pre-meiosis; M, meiosis II; E, early uninucleate; L, late uninucleate; G, gametogenesis; gDNA, genomic DNA. (PDF) [file pone.0177632.s002.pdf]

Supporting Information

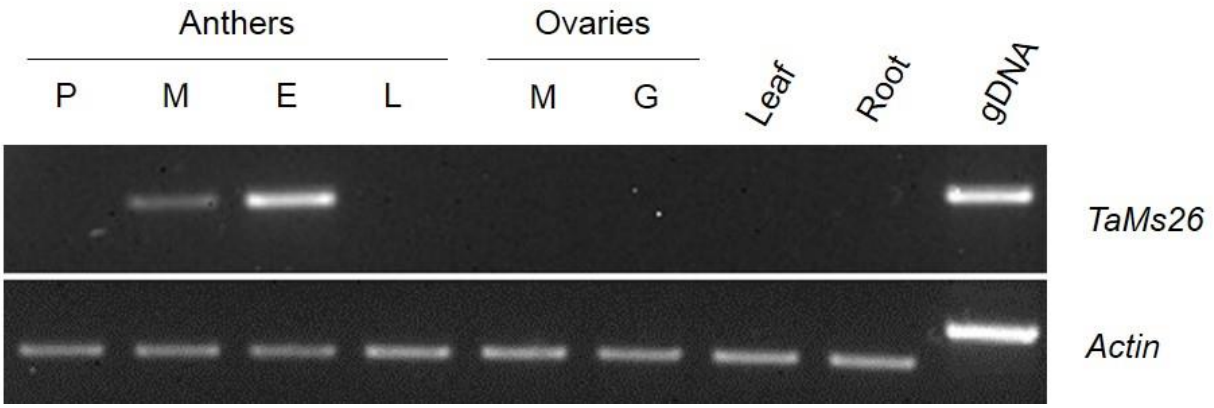

S2 Fig. Spatial and temporal expression of *TaMs26* by semi-quantitative RT-PCR. *TaActin* expression was used as a control. P, pre-meiosis; M, meiosis II; E, early uninucleate; L, late uninucleate; G, gametogenesis; gDNA, genomic DNA.
