# Supplementary material for: MS26/CYP704B is required for anther and pollen wall development in bread wheat (Triticum aestivum L.) and combining mutations in all three homeologs causes male sterility
Source: PLoS One. 2017 May 16;12(5):e0177632. doi: 10.1371/journal.pone.0177632 (PMC5433722; doi:10.1371/journal.pone.0177632)
Supplement: S3 Fig — Anther epidermis surface at late vacuolate microspore stage from wild type (A), Tams26-Aabd (B), Tams26-abDd (C), and Tams26-aBbd (D) plants. Scale bars = 20 μm. (PDF) [file pone.0177632.s003.pdf]

Supporting Information

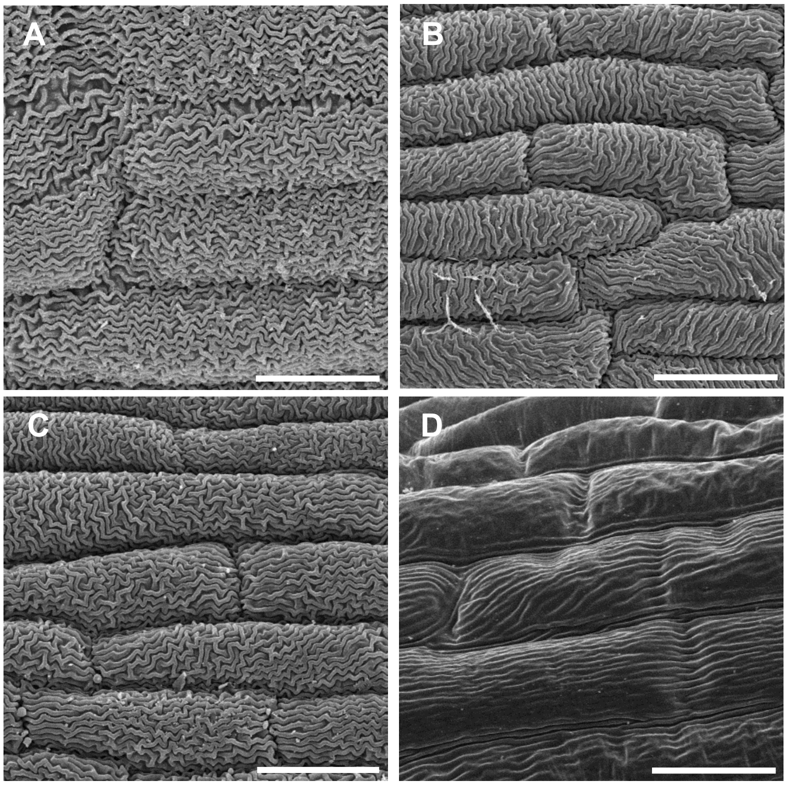

S3 Fig. Scanning Electron Microscopy of anther epidermis from double homozygous-single heterozygous *Tams26* mutant wheat plants. Anther epidermis surface at late vacuolate microspore stage from wild type (A), *Tams26-Aabd* (B), *Tams26-abDd* (C), and *Tams26-aBbd* (D) plants. Scale bars = 20 µm
