# Supplementary material for: MS26/CYP704B is required for anther and pollen wall development in bread wheat (Triticum aestivum L.) and combining mutations in all three homeologs causes male sterility
Source: PLoS One. 2017 May 16;12(5):e0177632. doi: 10.1371/journal.pone.0177632 (PMC5433722; doi:10.1371/journal.pone.0177632)
Supplement: S4 Fig — (PDF) [file pone.0177632.s004.pdf]

Supporting Information

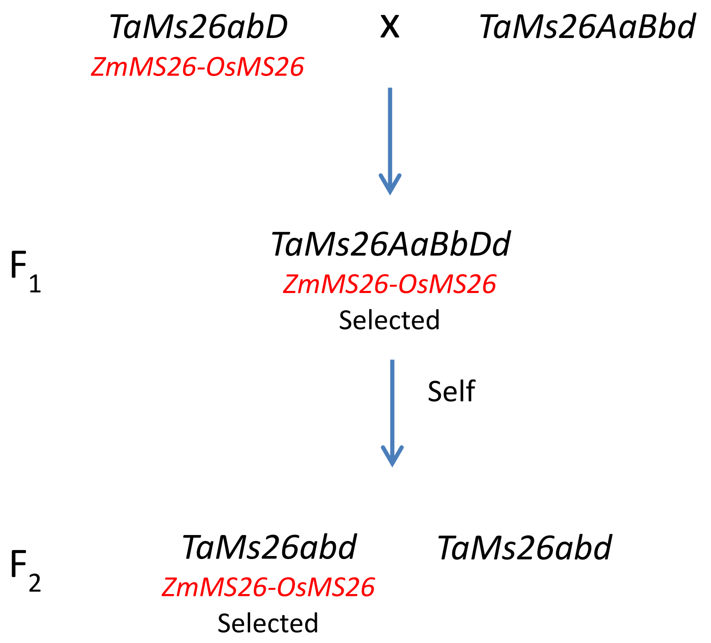

S4 Fig. Crossing strategy to combine wheat mutations with transformed maize and rice genes (red text) for complementation testing.
