# Supplementary material for: MS26/CYP704B is required for anther and pollen wall development in bread wheat (Triticum aestivum L.) and combining mutations in all three homeologs causes male sterility
Source: PLoS One. 2017 May 16;12(5):e0177632. doi: 10.1371/journal.pone.0177632 (PMC5433722; doi:10.1371/journal.pone.0177632)
Supplement: S1 Table — (PDF) [file pone.0177632.s005.pdf]

Supporting Information

S1 Table. List of primers used in the study.

Primer	Sequence[a]
UNIMS26 5’-2	GACGTGGTGCTCAACTTCGTGAT
UNIMS26 3’-1	GCCATGGAGAGGATGGTCATCAT
TAMS26-A	CGCGCTGGTCCCCTGCAGCGACTCAGAC
TAMS26-B	CATAACACGCCGGCCGTCTGGCCAGA
TAMS26-D	CCGGCCAGCCAGCCACCGGAGCGTCCAG
TAMS26-F	AGGGCATCGCGGAGGACGAC
TAMS26-R	CGCCTGGAACGCCGTGAACT
TAACTIN-F	TCCATCATGAAGTGCGACGTGGA
TAACTIN-R	TGCACGATGGATGGGCCAGA
*Ms26*F	CAAGCAGAAGACGGCATACGAGCTCTTCCGATCTCGCAACGCGTCGCCGTTCAA
*Ms26*R	CTACACTCTTTCCCTACACGACGCTCTTCCGATCTAACGTGGCCAGCGCCATCTTCATCTGCAGG
F2	AATGATACGGCGACCACCGAGATCTACACTCTTTCCCTACACG
R2	CAAGCAGAAGACGGCATA
OSMS26-F	TGTAGAATTGTGCCCTGCAATTTCTCAAATC
OSMS26-R	CCGAAGCTTCATCACCGCGTGCATCTGCTTGATGTGTC
OSMS26PRO-F	TGAAGGGTTCAGTCTACAAGAGATGC
OSMS26PRO-R	GCATTTCTTGGATTTGGGTGTTTGTAA
ZMMS26 TERM-F	TGTCATGGCGATTTGGATATGGATAT
ZMMS26 TERM-R	ATTTATAGATGTATACGGCGTCCCGG

[a] Primer orientation 5’ to 3’ is left to right.
